# Supplementary material for: Two-dimensional billiards are Turing complete
Source: Proc Natl Acad Sci U S A. 2026 Sep 10;123(37):e2614500123. doi: 10.1073/pnas.2614500123 (PMC13578349; doi:10.1073/pnas.2614500123)
Supplement: Supplementary file 1 [file pnas.2614500123.sapp.pdf]

RESEARCH ARTICLE | PHYSICAL SCIENCES/MATHEMATICS

Supplementary Appendix to the article *Two-Dimensional Billiards Are Turing Complete*

Eva Miranda[a,b,1,2] and Isaac Ramos[c,a,1]

This manuscript was compiled on August 6, 2026

The purpose of this appendix is to provide the missing details in the proof of Lemma 2 from the main text, thus completing the argument that the read–write operation can be realized by means of billiard dynamics. Namely, we give an explicit expression for the straight-segments section of the read–write wall. Then, we prove that the read–write dynamics are well-defined in the sense that after bouncing off the wall, any trajectory encoding a computational state does not collide again with any other part of the wall. Finally, we show that the wall can be made smooth by joining the straight segments smoothly in the gaps between them, except at the two accumulation points at the endpoints of the interval, where the wall can only be extended continuously.

We start by giving an explicit expression for the straight segments, the part of the read–write wall where the trajectories associated with computational states collide. Recall first that, according to our definition of a Turing machine, applying the read–write operation to a computation state (t, k) requires inspecting and possibly modifying the tape symbol t_k. Under our Cantor set–type encoding, this amounts to detecting and, if necessary, changing a specific digit in the ternary expansion associated with the tape before applying the embedding τ_k. For this reason, we argued that, inside the interval I_k corresponding to the head position k, the admissible computation states decompose into two alternating families of subintervals, depending on whether the symbol at position k is 0 or 1. We now make this decomposition explicit.

Fix $k \in \mathbb{Z}$ and, for simplicity, assume $k \geq 0$ (the case $k < 0$ is completely analogous). We begin by considering the encoding of the tape t alone as a point $x_t \in I$, prior to applying the embedding τ_k. Our goal is to describe two families of Cantor set blocks corresponding to the conditions $t_k = 0$ and $t_k = 1$. Recall that, in our encoding, the symbol t_k is stored in the $(2k+1)$-th digit of the ternary expansion of x_t. To simplify notation, we introduce

$$x_{\epsilon_1 \dots \epsilon_l} := 2 \sum_{n=1}^{l} \epsilon_n \, 3^{-n},$$

where each $\epsilon_n \in \{0, 1\}$.

With this notation, the Cantor set blocks corresponding to tapes with $t_k = 0$ are given by

$$I_{\epsilon_1 \dots \epsilon_{2k} 0} = \left[x_{\epsilon_1 \dots \epsilon_{2k} 0},\, x_{\epsilon_1 \dots \epsilon_{2k} 0} + 3^{-(2k+1)}\right], \qquad \epsilon_n \in \{0,1\},\ n = 1, \dots, 2k,$$

while those corresponding to tapes with $t_k = 1$ are

$$I_{\epsilon_1 \dots \epsilon_{2k} 1} = \left[x_{\epsilon_1 \dots \epsilon_{2k} 1},\, x_{\epsilon_1 \dots \epsilon_{2k} 1} + 3^{-(2k+1)}\right], \qquad \epsilon_n \in \{0,1\},\ n = 1, \dots, 2k.$$

We now apply the embedding τ_k, using its explicit expression from the proof of Lemma 1. Define

$$x^k_{\epsilon_1 \dots \epsilon_l} := \tau_k(x_{\epsilon_1 \dots \epsilon_l}) = 1 + 3^{-(1+k)}\left(-2 + x_{\epsilon_1 \dots \epsilon_l}\right).$$

Then the Cantor set blocks inside I_k corresponding to the condition $t_k = s$, with $s \in \{0, 1\}$, are

$$I^k_{\epsilon_1 \dots \epsilon_{2k} s} := \tau_k\left(I_{\epsilon_1 \dots \epsilon_{2k} s}\right) = \left[x^k_{\epsilon_1 \dots \epsilon_{2k} s},\, x^k_{\epsilon_1 \dots \epsilon_{2k} s} + 3^{-(3k+2)}\right],$$

again with $\epsilon_n \in \{0, 1\}$ for $n = 1, \dots, 2k$. There are 2^{2k} such intervals, each of length $3^{-(3k+2)}$.

We now describe the billiard walls implementing the read–write operation. We assume that incoming billiard trajectories move upward, with positive velocity in the y-direction, and have horizontal coordinate $x \in [0, 1]$, where the computation state (t, k) is encoded as $x_{t,k}$.

Author affiliations: [a]Laboratory of Geometry and Dynamical Systems and SYMCREA research unit, Department of Mathematics & IMTech,, Universitat Politècnica de Catalunya, Barcelona, Av. Dr Marañón 44-55, 08028, Barcelona, Spain ; [b]Centre de Recerca Matemàtica, CRM, Barcelona, Spain; [c]Department of Mathematics, ETH Zürich, Zürich, Switzerland

Author contributions: E.M. and I.R. designed the research, performed the research, and wrote the paper.

The authors declare no competing interests

[1]E.M.(Author One) contributed equally to this work with I.R. (Author Two) .

[2]To whom correspondence should be addressed. E-mail: eva.miranda@upc.edu

We first treat the case in which no symbol change occurs, so that the goal is simply to distinguish between $t_k = 0$ and $t_k = 1$.

After interacting with the billiard walls, we want trajectories corresponding to $t_k = 0$ to continue vertically with horizontal coordinate $x_{t,k} - 2$, while those corresponding to $t_k = 1$ should continue vertically with horizontal coordinate $x_{t,k}+2$. To achieve this separation, trajectories first collide with straight walls placed above the intervals $I^k_{\epsilon_1\ldots\epsilon_{2k}s}$, with slope -1 for $s = 0$ and slope $+1$ for $s = 1$.

For $t_k = 0$, we define the walls

$$W^k_{\epsilon_1\ldots\epsilon_{2k}0}(x) = 2+x^k_{\epsilon_1\ldots\epsilon_{2k}0}+\frac{3^{-(3k+2)}}{2}-x, \qquad x \in I^k_{\epsilon_1\ldots\epsilon_{2k}0}.$$

Each such wall is a line segment of slope -1, centered horizontally above the corresponding interval $I^k_{\epsilon_1\ldots\epsilon_{2k}0}$ and located at height $y = 2$.

Similarly, for $t_k = 1$, we take walls of slope $+1$ given by

$$W^k_{\epsilon_1\ldots\epsilon_{2k}1}(x) = 2-x^k_{\epsilon_1\ldots\epsilon_{2k}1}-\frac{3^{-(3k+2)}}{2}+x, \qquad x \in I^k_{\epsilon_1\ldots\epsilon_{2k}1}.$$

These walls deflect trajectories corresponding to $t_k = 0$ to the left and those corresponding to $t_k = 1$ to the right.

Once the trajectories have been separated, we must redirect them so that they again travel vertically. For instance, in the case $t_k = 0$, this is achieved by introducing a second family of walls

$$\widetilde{W}^k_{\epsilon_1\ldots\epsilon_{2k}0}(x) = x^k_{\epsilon_1\ldots\epsilon_{2k}0} + \frac{3^{-(3k+2)}}{2} - (x + 2),$$

defined for

$$x \in \left[x^k_{\epsilon_1\ldots\epsilon_{2k}0} - 2,\, x^k_{\epsilon_1\ldots\epsilon_{2k}0} - 2 + 3^{-(3k+2)}\right].$$

A trajectory starting at $x_{t,k}$ with $t_k = 0$ first reflects off the wall $W^k_{\epsilon_1\ldots\epsilon_{2k}0}$, then travels along a line of slope -1 until it reaches $\widetilde{W}^k_{\epsilon_1\ldots\epsilon_{2k}0}$. Since the two walls are parallel, the outgoing trajectory is vertical, with horizontal coordinate $x_{t,k} - 2$, as desired (see Figure 1). A similar construction can be used to have the outgoing trajectory corresponding to $t_k = 1$ travel vertically with horizontal coordinate $x_{t,k} + 2$.

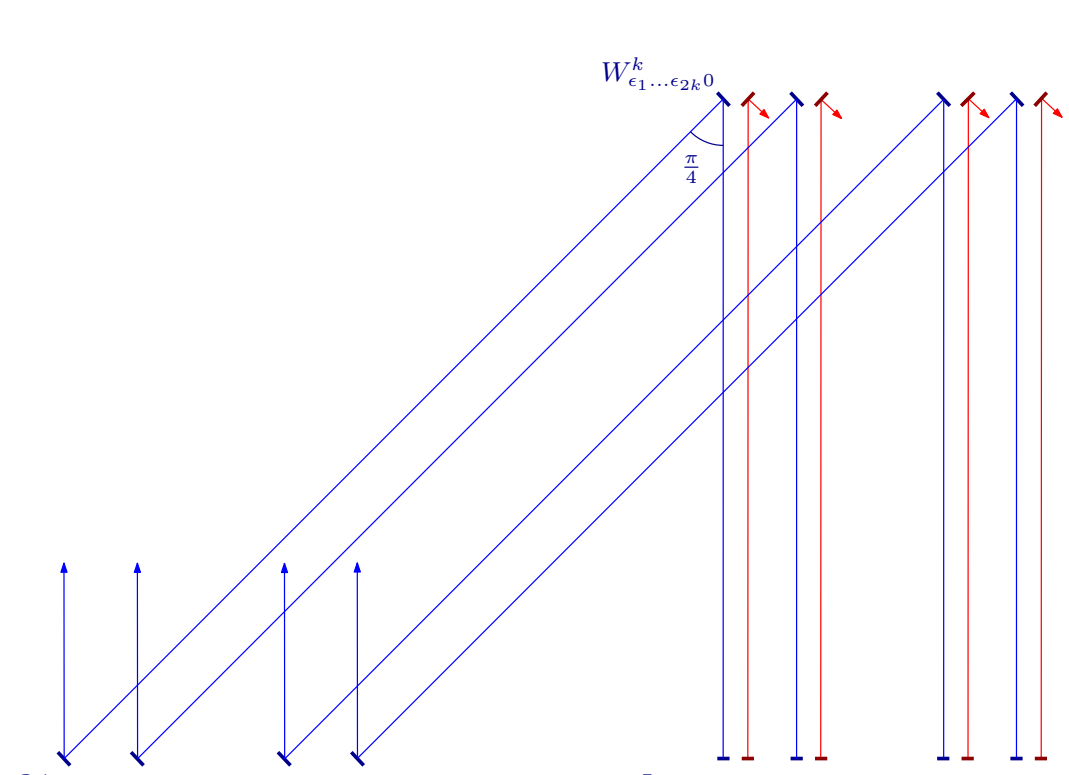

Fig. 1. Billiard dynamics implementing the read–write operation with no symbol change. The blue and red rays correspond to reading a 0 or a 1, respectively.

We now turn to the case in which a symbol change is required. For simplicity, suppose that we want to modify the tape symbol at position k from $t_k = 0$ to $t'_k = 1$. In terms of our encoding, this means that an incoming vertical trajectory with horizontal coordinate $x_{t,k}$ should be transformed, after interacting with the read–write construction, into a vertical trajectory with horizontal coordinate

$$x_{t,k} + 2 \cdot 3^{-(3k+2)} - 2.$$

Compared with the pure read case, this requires a slight horizontal displacement of the trajectory. Therefore, the separating wall can no longer have slope exactly -1. Instead, we choose the angle α_k with the horizontal so that

$$\tan \alpha_k = \frac{1}{1 + 3^{-(3k+2)}}.$$

This deviation from slope -1 is very small, especially for large values of k, and in the limit $k \to \infty$ we recover the original configuration. We can observe this in Figure 2, where the small displacement is displayed compared to the original no-rewrite wall.

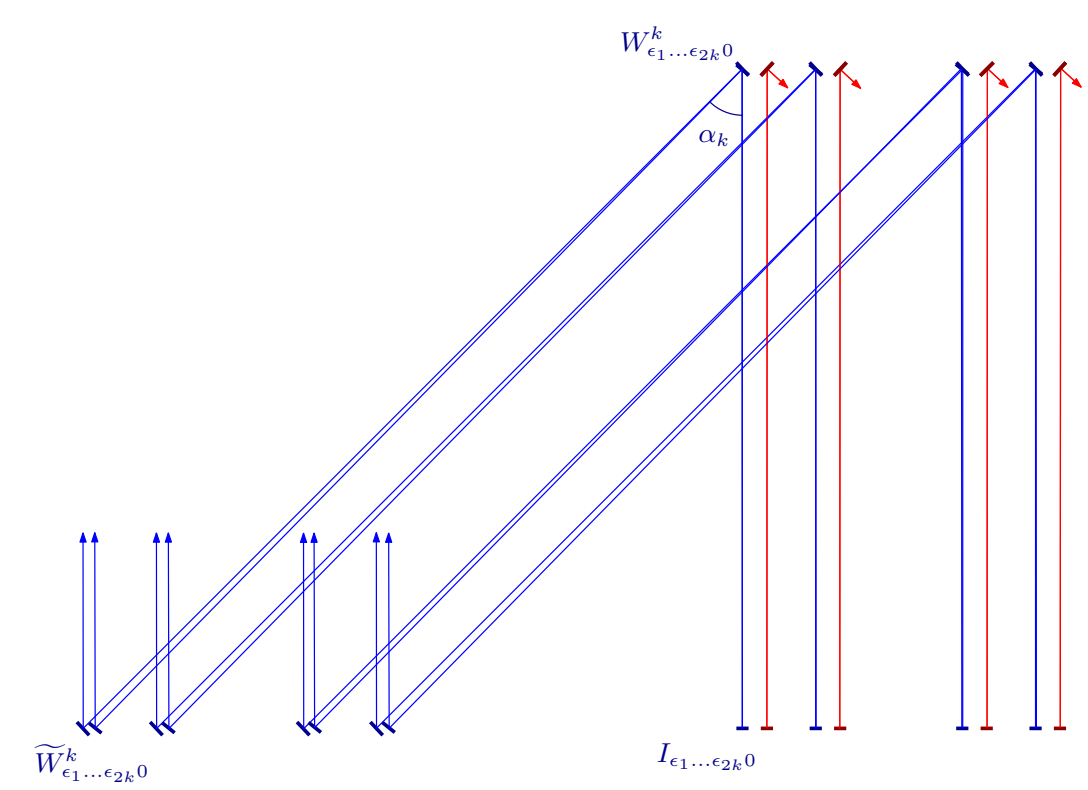

Fig. 2. Billiard dynamics implementing the read–write operation with symbol change compared to the no-change situation.

We can now prove that the read–write operation can be performed with these walls, and in particular, that the corresponding trajectories do not collide with any other wall from this construction.

Lemma 1. *Let us consider any $k \in \mathbb{Z}$ (we can assume $k \geq 0$), $t \in \Lambda$ and $s \in \{0, 1\}$. Assume that $x_{t,k} = x^k_{\epsilon_1\ldots\epsilon_{2k}t_k}$ for some $\epsilon_1 \ldots \epsilon_{2k} \in \{0, 1\}$. We claim that the billiard trajectory starting at $(x^k_{\epsilon_1\ldots\epsilon_{2k}t_k}, 0)$ with vertical direction, travels from the wall $W^k_{\epsilon_1\ldots\epsilon_{2k}t_k}$ to the corresponding $\widetilde{W}^k_{\epsilon_1\ldots\epsilon_{2k}s}$ without colliding with any other wall of the construction.*

Proof. First, since all walls lie at the same height, this reduces to checking that, after bouncing off $W^k_{\epsilon_1\ldots\epsilon_{2k}s}$, the trajectory descends sufficiently fast so as not to intersect any other wall $W^{k'}_{\epsilon'_1\ldots\epsilon'_{2k'}s'}$.

Observe that trajectories corresponding to a symbol change have a steeper downward slope than those in the no-rewrite case. As a result, avoiding collisions is strictly easier in the rewriting case. It therefore suffices to carry out the check for the read–only configuration, where the walls have slopes ± 1.

Consider a trajectory that bounces off a wall $W^k_{\epsilon_1\ldots\epsilon_{2k}0}$, i.e., the case $t_k = 0$. Until it reaches $\widetilde{W}^k_{\epsilon_1\ldots\epsilon_{2k}0}$, this trajectory remains parallel to all walls of the form

$W^{k'}_{\epsilon'_1\ldots\epsilon'_{2k'}1}$ (or their slope is larger when the symbol 1 is modified by the transition). Since such walls are separated by a horizontal distance of at least $2\cdot 3^{-(3k+2)}$, there is no risk of collision with them. We therefore only need to check that the trajectory does not hit any wall $W^{k'}_{\epsilon'_1\ldots\epsilon'_{2k'}0}$ lying to its left. It is enough to consider the extremal situation. Namely, we check that the trajectory bouncing off the left endpoint of $W^k_{\epsilon_1\ldots\epsilon_{2k}0}$, located at

$$\left(x^k_{\epsilon_1\ldots\epsilon_{2k}0},\, 2+3^{-(3k+2)}/2\right),$$

does not intersect the right endpoint of any wall $W^{k'}_{\epsilon'_1\ldots\epsilon'_{2k'}0}$, located at

$$\left(x^{k'}_{\epsilon'_1\ldots\epsilon'_{2k'}0}+3^{-(3k'+2)},\, 2-3^{-(3k'+2)}/2\right).$$

This amounts to verifying the inequality

$$x^k_{\epsilon_1\ldots\epsilon_{2k}0}-\left(x^{k'}_{\epsilon'_1\ldots\epsilon'_{2k'}0}+3^{-(3k'+2)}\right)>\frac{3^{-(3k+2)}}{2}+\frac{3^{-(3k'+2)}}{2}.$$

We first consider the case $k=k'$. By construction, if $x^k_{\epsilon_1\ldots\epsilon_{2k}0}\neq x^k_{\epsilon'_1\ldots\epsilon'_{2k}0}$, then

$$x^k_{\epsilon_1\ldots\epsilon_{2k}0}-x^k_{\epsilon'_1\ldots\epsilon'_{2k}0}\geq 2\cdot 3^{-(3k+1)}.$$

It follows that

$$x^k_{\epsilon_1\ldots\epsilon_{2k}0}-\left(x^k_{\epsilon'_1\ldots\epsilon'_{2k}0}+3^{-(3k+2)}\right)\geq 3^{-(3k+2)}(6-1)>3^{-(3k+2)},$$

which is more than enough to guarantee the desired separation.

We now turn to the case $k\neq k'$. In this situation, $x^k_{\epsilon_1\ldots\epsilon_{2k}0}\in I_k$ and $x^{k'}_{\epsilon'_1\ldots\epsilon'_{2k'}0}\in I_{k'}$. Since the distance between $I_{k'}$ and $I_{k'+1}$ is $3^{-(k'+2)}$, the distance between $I_{k'}$ and I_k is at least this large. Consequently,

$$\begin{aligned}x^k_{\epsilon_1\ldots\epsilon_{2k}0}-\left(x^{k'}_{\epsilon'_1\ldots\epsilon'_{2k'}0}+3^{-(3k'+2)}\right)&\geq 3^{-(k'+2)}\\ &\geq 3^{-(3k'+2)}>\frac{3^{-(3k+2)}}{2}+\frac{3^{-(3k'+2)}}{2}.\end{aligned}$$

where in the last inequality we used the fact that $k>k'$.

We conclude that, after bouncing off $W^k_{\epsilon_1\ldots\epsilon_{2k}0}$, the trajectory does not intersect any other wall $W^{k'}_{\epsilon'_1\ldots\epsilon'_{2k'}s'}$. By symmetry, the same argument applies to walls of the form $W^k_{\epsilon_1\ldots\epsilon_{2k}1}$ and to possible collisions with $\widetilde{W}^k_{\epsilon_1\ldots\epsilon_{2k}s}$, as well as to the case $k<0$. □

We now explain how to join the straight segments so as to obtain a wall with the desired regularity that implements the read–write operation.

Proposition 1. *There exists a connected wall implementing the read–write operation through billiard dynamics. This wall is smooth away from its endpoints, where it extends only continuously.*

Proof. First, we note that the estimates used above to rule out unwanted collisions are far from sharp, meaning there is a positive margin of safety. This allows us to create a smooth wall by joining the straight segments with smooth arcs inside the gaps between them. Since no computational state is encoded in these gaps, the wall can be modified there without affecting the simulated computation, provided the smooth interpolations remain sufficiently close to the linear segments to preserve the non-collision estimates. This smoothing can be performed cleanly everywhere except at the two limit points corresponding to $k\to\pm\infty$. Indeed, our construction is locally finite: any compact subinterval away from the endpoints intersects only finitely many intervals I_k, and since each I_k contains a finite number of straight segments, any such subinterval contains only finitely many oscillations. Thus, we can remove every interior singularity, rendering the wall C^∞-smooth everywhere except at the two endpoint accumulation points, where any neighborhood necessarily contains infinitely many oscillations.

It remains to analyze the behavior of the wall near these two endpoints. Although the smoothing procedure cannot be extended there, the wall still admits a well-defined continuous extension. Indeed, let us consider the portion of the wall corresponding to a fixed interval I_k and consider the case $k\geq 0$,i.e., the limit point on the right corresponding to $k\to\infty$ (the case $k\to-\infty$ is analogous). The oscillatory linear segments of this portion are confined to the Cantor blocks of order $2k+1$. Since there are 2^{2k} such blocks, each of length $3^{-(2k+1)}$, their total length is

$$2^{2k}3^{-(2k+1)}=\frac{1}{3}\left(\frac{4}{9}\right)^k\xrightarrow[k\to\infty]{}0.$$

Moreover, each zig–zag segment has vertical size on the order of $3^{-(3k+2)}$, which also tends to zero as $|k|\to\infty$. Therefore, both the horizontal support and the vertical amplitude of the oscillations vanish near the endpoints of the interval. It follows that the restriction of the wall to I_k converges uniformly to the straight segment joining the endpoints of I_k. Consequently, the entire billiard wall extends continuously to the two accumulation points, although these remain the only points where the boundary is not smooth. Indeed, as one approaches either endpoint, the wall still contains infinitely many linear portions with slopes alternating between 1 and -1 (or slopes asymptotically approaching these values, if there is a symbol change). Consequently, the tangent direction oscillates indefinitely and does not admit a limit at either accumulation point. Therefore, the present construction cannot be extended to a C^1 curve there. □

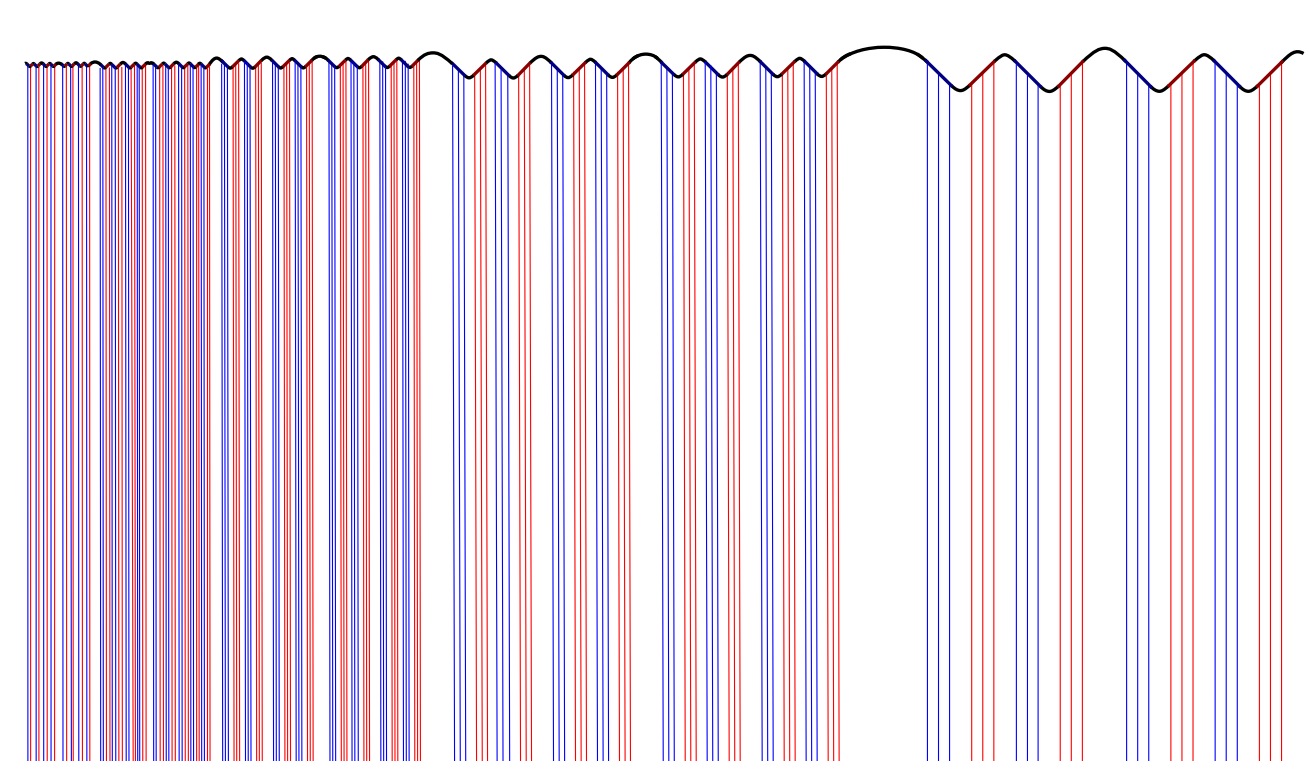

Fig. 3. How the billiard wall implementing the read–write operation changes as $k\to-\infty$.
